# *Nahuatlea*: a new genus of compositae (Gochnatieae) from North America

**DOI:** 10.3897/phytokeys.91.21340

**Published:** 2017-12-18

**Authors:** Vicki A. Funk, Gisela Sancho, Nádia Roque

**Affiliations:** 1 US National Herbarium, Department of Botany, Smithsonian Institution – NMNH, MRC 166, Washington DC, 20560, USA; 2 Museo de La Plata, Paseo del Bosque s/n, 1900 La Plata, BA, Argentina; 3 Instituto de Biologia, Universidade Federal da Bahia, Campus Universitário de Ondina, 40170-110 Salvador, Bahia, Brazil

**Keywords:** Asteraceae, *Gochnatia*, Gochnatioideae, Mexico, Texas, Arizona, South American-Mexican disjunct distribution

## Abstract

In the course of a detailed molecular study of the tribe Gochnatieae (Compositae: Gochnatioideae) it became apparent that the genus *Gochnatia* (sensu Cabrera) was not monophyletic but composed of a number of morphologically, geographically, and molecularly distinct clades. All but one of these clades had previously been recognized at the generic or sectional level and therefore had a name that could be applied. However, one clade, whose members are from Mexico and adjacent parts of the United States, had never been recognized as a distinct taxon. The Mexican clade is the sister group of the Caribbean clade which seems to indicate a dispersal event from Southern South America to Mexico and from Mexico to the Caribbean. Here we provide the Mexican clade with a genus name, *Nahuatlea*, and make the necessary new combinations. The name is derived from Nahuatl, the major indigenous language that is spoken in the area where most of the collections were gathered. A genus description, key to species, images, and a short, species descriptions, are provided.

## Introduction

**Figure 1.**
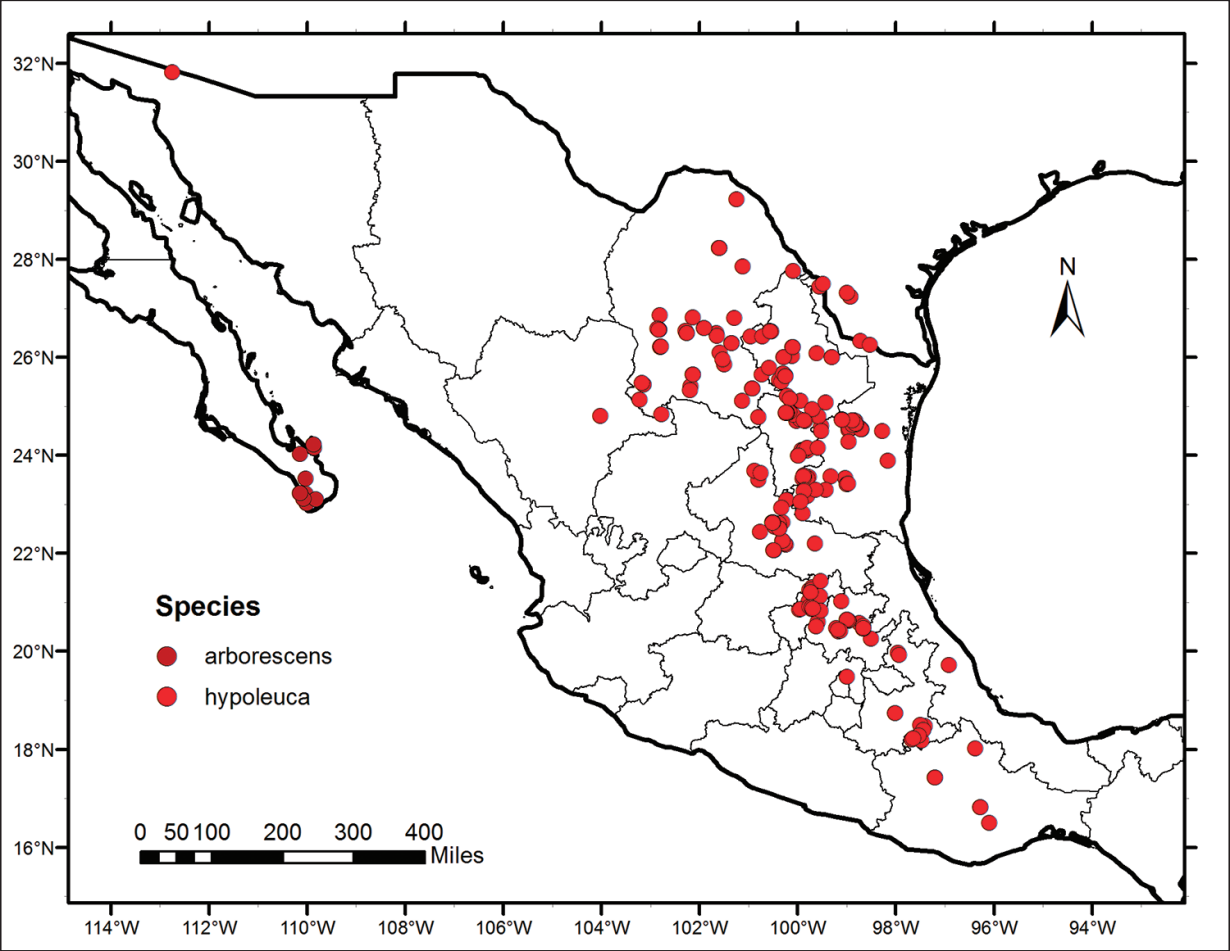
Distribution map of *Nahuatlea arborescens* and *N. hypoleuca*.

**Figure 2.**
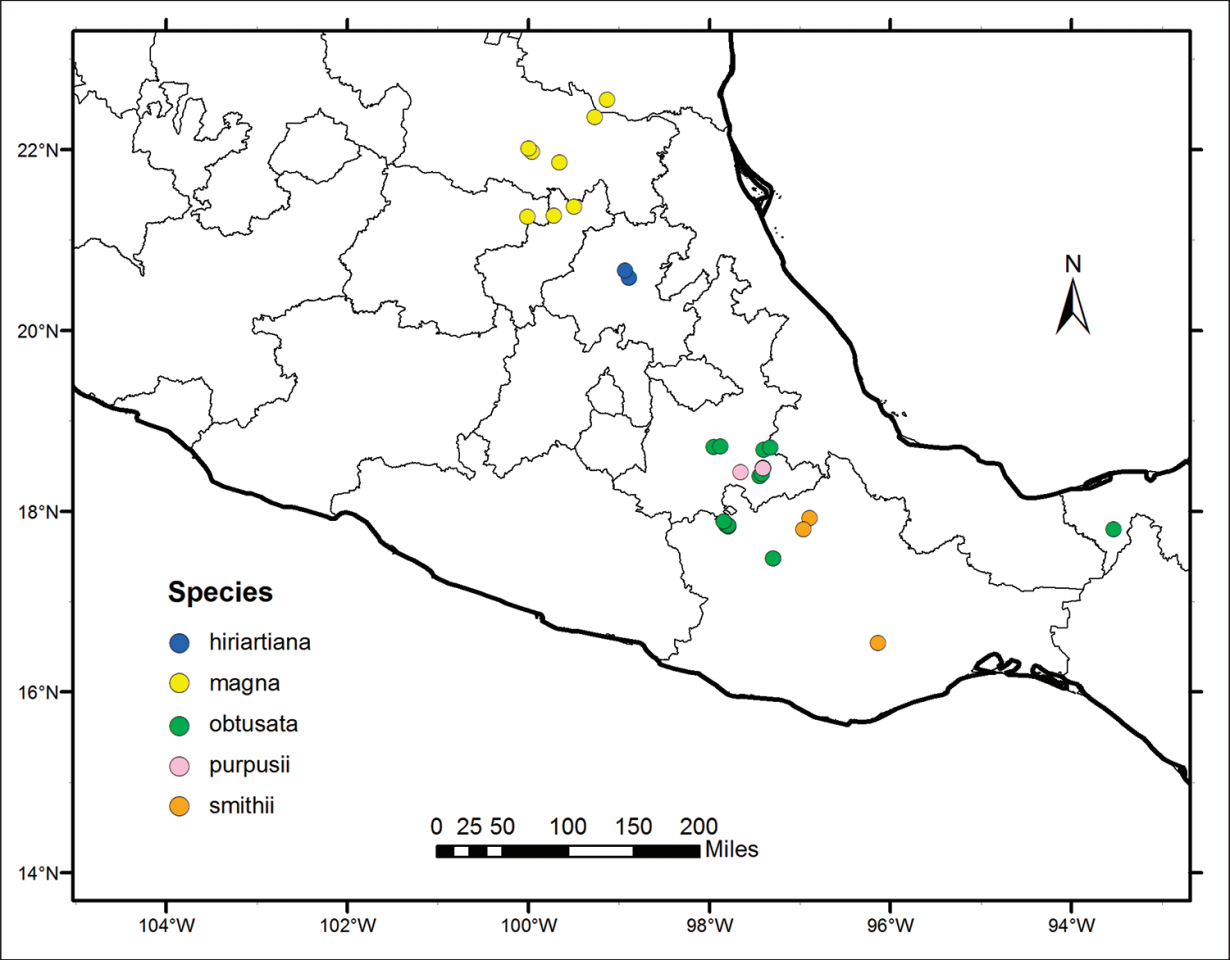
Distribution map of *Nahuatlea hiriartiana*, *N. magna*, *N. obtusata*, *N. purpusii*, and *N. smithii*.

Cabrera (1971) in his comprehensive treatment of *Gochnatia* Kunth recognized six sections in the genus all mainly from the Americas. He placed all known species of *Gochnatia* from Mexico, in the section Hedraiophyllum (Less.) DC. along with two southern South American species: *G. palosanto* Cabrera and *G. cordata* Less., which are now part of *Gochnatia* s.s. and *Moquiniastrum, r*espectively. Although one species from Mexico is rather widespread (it has been collected from a number of states in Mexico, a few counties in Texas, and there is one disjunct record from Arizona) all of the species can be found in Mexico (Figs 1, 2).

Cabrera recognized five species of *Gochnatia* from Mexico and adjacent USA: *G. arborescens* Brandegee, *G. hypoleuca* (DC.) A. Gray, *G. magna* M.C. Johnson ex Cabrera, *G. purpusii* Brandegee, and *G. smithii* Robins. & Greenm. *Gochnatia obtusata* S.F. Blake, although accepted by Jervis (1954), was not accepted as a species by Cabrera (1971), instead it was placed as subspecies under the widespread *G. hypoleuca*. According to data from molecular studies (Funk et al. 2014), Gochnatia section Hedraiophyllum (Cabrera 1971) is not supported but it is interesting to note that Cabrera considered the section Hedraiophyllum to be somewhat artificial.

The next comprehensive treatment of *Gochnatia* was by Freire et al. (2002). In this treatment, the authors placed 21 species from South America in section Hedraiophyllum but not the Mexican species as Cabrera did (1971); in fact, their concept of this section was very different from Cabrera’s (1971). Freire et al. (2002) placed five species in Gochnatia sect. Leucomeris (D. Don) Cabrera, two of which were from Mexico: *G. hypoleuca* (including *G. obtusata*) and *G. smithii*. The other three species in G. sect. Leucomeris were distributed in Brazil, the Andean region of Bolivia and Argentina, and southeastern Asia. Freire et al. (2002) described a new section, G. sect. Glomerata S. E. Freire, L. Katinas & G. Sancho, consisting entirely of the final three Mexican species: *G. arborescens*, *G. magna*, *G. purpusii*. Freire et al. (2002) placed the Caribbean species (now in *Anastraphia*) in section Anastraphioides Jervis ex S.E. Freire, L. Katinas & G. Sancho. The molecular based study of Funk et al (2014) did not support the arrangement of taxa as proposed by Freire et al. (2002) except for section Anastraphioides (the Caribbean species) which formed a strongly supported clade.

In 2004 González-Medrano et al. described a new species of *Gochnatia* from Hidalgo, Mexico: *G. hiriartiana* Medrano, Villaseñor & Medina. It differs from all the other Mexican species because of its few, large heads, and greater number of disc flowers. The authors included a key to seven taxa: six species and the subspecies G. hypoleuca subsp. obtusata. The authors placed the new species in G. sect. Glomerata based on the key in Freire et al. (2002). It seems that González-Medrano et al. (2004) are the only authors to refer to the “Mexican species of *Gochnatia*” but they left them in two groups in different sections and never explicitly said that they thought the taxa formed a closely related group.

The recent molecular study of the tribe Gochnatieae (Funk et al. 2014) showed eight well-supported clades: most species had previously been included in the large genus *Gochnatia*.

All but one of the clades had previously been recognized at the generic or subgeneric level:

1) *Gochnatia* Kunth: Eight species from the Central Andes (more or less equal to G. sect. Gochnatia of Freire et al. 2002)

2) *Pentaphorus* D. Don: Southern Andean clade of two species that, at various times, had been recognized as a genus, subgenus, or section (resurrected by Hind 2007).

3) Mexican *Gochnatia*: A group of seven species that had never been recognized as a separate genus nor had they been placed in a section by themselves was recovered as a monophyletic group.

4) *Anastraphia*: The 33 Caribbean species fell into one strongly supported clade that had previously been recognized as a genus and a section (re-established as a genus by Ventosa and Herrera 2011a; Robinson and Funk 2012).

5) *Moquiniastrum*: The 21 species that form this clade are mainly from central and southern Brazil and previously formed the majority of Gochnatia sect. Moquiniastrum (Sancho et al. 2013).

6) *Richterago* Kuntze: A clade of 16 species, all endemic to the campos rupestres of Brazil (redefined by Roque and Pirani 2001, 2014).

7) *Cnicothamnus* Griseb.: A genus of two species found in Bolivia and northwestern Argentina: it has never been included in *Gochnatia*.

8) *Cyclolepis* Gilles ex D. Don: A monospecific genus that has never been included in *Gochnatia*: found from Paraguay to the northern Patagonia area of Argentina. This lineage was regarded as “incertae sedis” in Funk et al. (2014) and so it remains.

A key to the genera of Gochnatieae along with additional discussion on the tribe is contained in a upcoming paper (Funk et al. in prep).

In the Funk et al. biogeographic analysis (2014), it was hypothesized that the tribe had a southern South America origin (including the Central and Southern Andes, Argentina, and southern Brazil). Based on this, plus the fact that the Mexican clade is the sister group of the Caribbean clade (*Anastraphia*) suggests, or is best explained by, a dispersal event from Southern South America to Mexico and from Mexico to the Caribbean. We order the dispersal events in this direction because the Mexican *Gochnatia* are morphologically more similar to the Andean genus *Gochnatia* s.s. than the Caribbean species. This Mexican + Caribbean clade (MCC) is believed to have separated from the rest of the Gochnatieae between 11 and 23 mya at a time when there was a gradual climate warming (early Miocene; Petuch 2003, Zachos et al. 2001). MCC is the sister group of a large clade containing *Richterago* + *Moquiniastrum* (RM) which is mainly from Brazil and Southern South America. The sister groups to these two large clades (MCC + RM) are from the Andes (*Gochnatia* s.s., *Pentaphorus*, *Cnicothamnus*), so just where in southern South America the MCC clade originated is uncertain especially since some of the sister group relationships are not well supported, although all of the segregate genera are individually clearly monophyletic.

The Mexican members of *Gochnatia* clearly do not belong in the genus *Gochnatia* s.s., therefore, we are here describing the genus *Nahuatlea* for these seven species.

## Materials and methods

**Figure 3.**
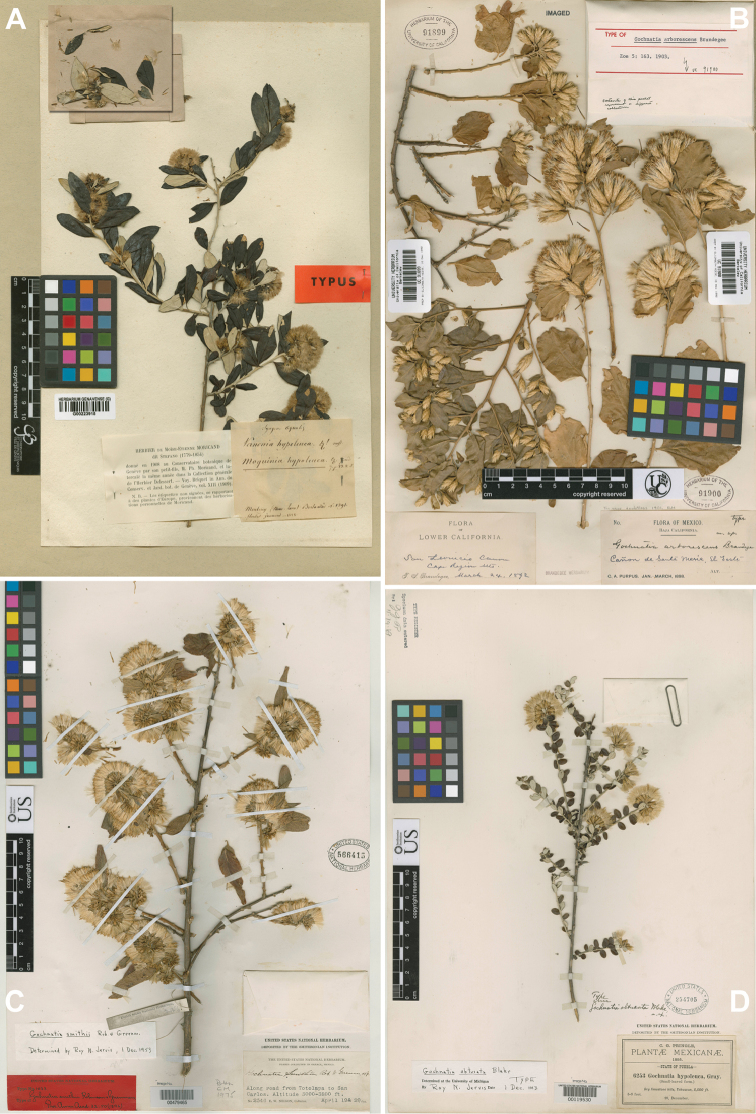
Images of four type specimens: **A** *Nahuatlea arborescens* (UC holotype) **B** *N. smithii* (K lectotype) **C** *N. obtusata* (US holotype) **D** *N. hypoleuca* (G holotype). [Furnished by the respective herbaria.]

All types and other specimens deposited at LP and US were examined. The remaining types were viewed on line using the JSTOR-GP portal (http://plants.jstor.org; Fig. 3).

Distributions are based on data derived from databasing and georeferencing the collections at US (available online http://collections.mnh.si.edu/search/botany/) with additional records from ARIZ (http://ag.arizona.edu/herbarium/home), MEXU (http://unibio.unam.mx/minero/index.jsp?accion=sc&colecciones=MEXU,Herbario), MO (http://www.tropicos.org/Home.aspx), TEX (http://orchid.biosci.utexas.edu/Texas_list.html), and NY (http://sciweb.nybg.org/science2/vii2.asp). When possible, records from GBIF (http://www.gbif.org/occurrence) were also added. Figures 1 and 2 were produced using ArcGIS v.10.2 (ESRI 2011).

## Taxonomy

### Nahuatlea

Taxon classificationPlantaeAsteralesCompositae

V. A.Funk

urn:lsid:ipni.org:names:60475585-2

#### Description.

*Shrubs* or trees, monoecious. *Leaves* sessile or with a short petiole of no more than 5 mm long, alternate, chartaceous or coriaceous, entire, margins revolute, usually discolorous (silvery or cinereous-tomentose beneath), clustered near the ends of the branches especially late in the flowering cycle. Heads arranged in clusters, rarely solitary, most branches with an apical cluster; sessile or short-pedunculate, peduncles commonly densely scaly-bracted resembling the lowermost involucral bracts, homogamous (flowers bisexual), discoid; involucre obconic (turbinate) or campanulate, shorter than the flowers; phyllaries imbricate, 4–10 seriate, graduate, coriaceous or subcoriaceous. *Flowers* with corollas homomorphic, white or yellow, actinomorphic, deeply 5-lobed, lobes equal or shorter than the tube, revolute; anthers calcarate, caudate, anther apical appendages apiculate, tails 1–3 mm long, entire or fimbrillate; styles rounded at apex, glabrous, style branches concave. *Achenes* 5-ribbed sericeous, cuneate-cylindrical, carpopodium conspicuous. *Pappus* of scabrid bristles, uni- or biseriate, graduated (varying in length) and equally wide throughout or rarely flattened at the tips, ca 1 cm long. [7 species]

#### Remarks.

*Nahuatlea*, with some exceptions, is characterized by a combination of characters: short leafy branchlets, entire and revolute blade margins, clusters of sessile or short-pedunculate heads at or near the tips of branches; densely scaly-bracted peduncles (when present); and a pappus that is biseriate, graduated, and equally wide throughout. Most of the exceptions are found in the recently described *Gochnatia hiriartiana* (Medrano, Villasenor & Medina, 2004) which has solitary heads, and a uniseriate pappus with bristles that are flattened at the tips. However, recently produced sequence data including those of *G. hiriartiana* support *Nahuatlea* as monophyletic (Funk, unpublished).

#### Etymology.

The genus name was selected to honor the indigenous people of eastern central Mexico by naming it after their language. The name is derived from *Nahuatl*, a language of the *Nahuan* branch of the Uto-Aztecan language family (known informally as Aztec). It is spoken by an estimated 1.5 million people, most of whom live in Central Mexico. *Nahuatl* has been spoken in Central Mexico since at least the 7th century AD and it was the language of the Aztecs who dominated what is now central Mexico during the Late Postclassic period of Mesoamerican history. Today the *Nahuatl* language is spoken in the Mexican states of Durango, Guerrero, Hidalgo, Mexico, Michoacan, Morelos, Oaxaca, Puebla, Tlaxcala, and Veracruz. The distribution of the new genus, *Nahuatlea*, in central Mexico has substantial overlap with the area so it is appropriate to use it for the name of the new genus (http://en.wikipedia.org/wiki/Nahuatl).

##### Key to species of *Nahuatlea*

**Table:** 

1	Involucre campanulate or broadly obconic, bracts wooly tomentose or puberulous abaxially; corollas yellow	**2**
–	Involucre narrowly cylindrical or narrowly obconic, bracts glabrous (may be ciliolate); corollas white or yellow (the latter only in *N. arborescens*)	**4**
2	Heads with 200–230 flowers; involucre 25–35(–40) mm in diameter; corolla 13–22 mm long	***N. hiriartiana***
–	Heads with 12–50 flowers; involucre 5–12 mm in diameter; corolla 8–12 mm long	**3**
3	Leaves 5–10 × 4–6.5 cm; involucre 10–12 mm wide; heads with ca. 50 flowers; 3–5 heads per cluster; pappus 9 mm long, bristles uni-seriate ; shrub or small tree 2.5–4.5 meters	***N. magna***
–	Leaves 1.8–3.0 × 0.5–1.4 cm; involucre 5–6 mm wide; heads with 12–18 flowers; 10–15 heads per cluster; pappus 7.5 mm long, bi-seriate; shrub (no size given)	***N. purpusii***
4	Leaves glabrescent abaxially; involucral bracts in 8–10 series; heads 6–8 mm wide; 13–20 flowers per head, corollas yellowish, 12 mm long; trees of 3–8 m, trunks 20–30 cm in diameter	***N. arborescens***
–	Leaves tomentose abaxially; involucral bracts in 3–7 series (bracts on peduncle not included); heads ca. 3 mm wide; (3–)5–7 flowers per head, corollas white, 5–12 mm long; shrubs or small trees of 1–7 m, stems less than 10 cm in diameter	**5**
5	Leaves 4–8 mm × 3–5 mm, broadly ovate; leaves at the glomerule shorter than the heads; involucral bracts usually glabrous	***N. obtusata***
–	Leaves 25–50 mm × 15–20 mm, oblong-lanceolate; leaves at the glomerule longer than the heads; involucral bracts ciliolate or ciliolate-tomentose on the margins	**6**
6	Involucral bracts in 5–7 series; corollas 8.5–9 mm long; anther tails entire; involucre 6–8 mm tall; shrub; blade oblong-lanceolate, apex acute, tomentose below	***N. smithii***
–	Involucral bracts in 3–5 series; corollas 7–12 mm long; anther tails fimbrillate; involucre 4–7 mm tall; shrub to small tree; blade narrowly elliptic, apex rounded, mucronate, whitish-tomentose below	***N. hypoleuca***

### 1. Nahuatlea hiriartiana

Taxon classificationPlantaeAsteralesCompositae

(Medrano, Villaseñor & Medina) V.A.Funk comb. nov.

urn:lsid:ipni.org:names:60475586-2

Fig. 4

Gochnatia hiriartiana Medrano, Villasenor & Medina. Novon 14: 435–436. 2004.

#### Type.

Mexico. Hidalgo: Municipio Meztitlán, 3 km al E de Milpa Grande, barranca sobre el Río Amajac, 19 Sep 1996, *F. González-Medrano, G.G. Hernández & G. Rodriguez 17920* (Holotype: MEXU 00316791; Isotypes, IEB 000177217, MO 3024215, TEX 00208274, XAL 0106702).

#### Description.

*Shrub* 1.0–1.5 m tall; *leaves* coriaceous, blades 1.0–3.5 × 1–2 cm, elliptic to slightly ovate, yellowish-tomentose below; heads sessile, solitary or in clusters of 2–3 at apex of branches, few clusters per plant; *involucre* campanulate, 15–20 mm tall × 25–35(–40) mm wide, bracts in 5–6 series, densely puberulous abaxially; *flowers* 200–230 per head; corollas light yellow, 13–22 mm long; *anther* base caudate, tails 2.0–2.5 mm long, entire; *pappus* ca. 1 cm long, bristles biseriate, broadening slightly at the apex.

**Figure 4.**
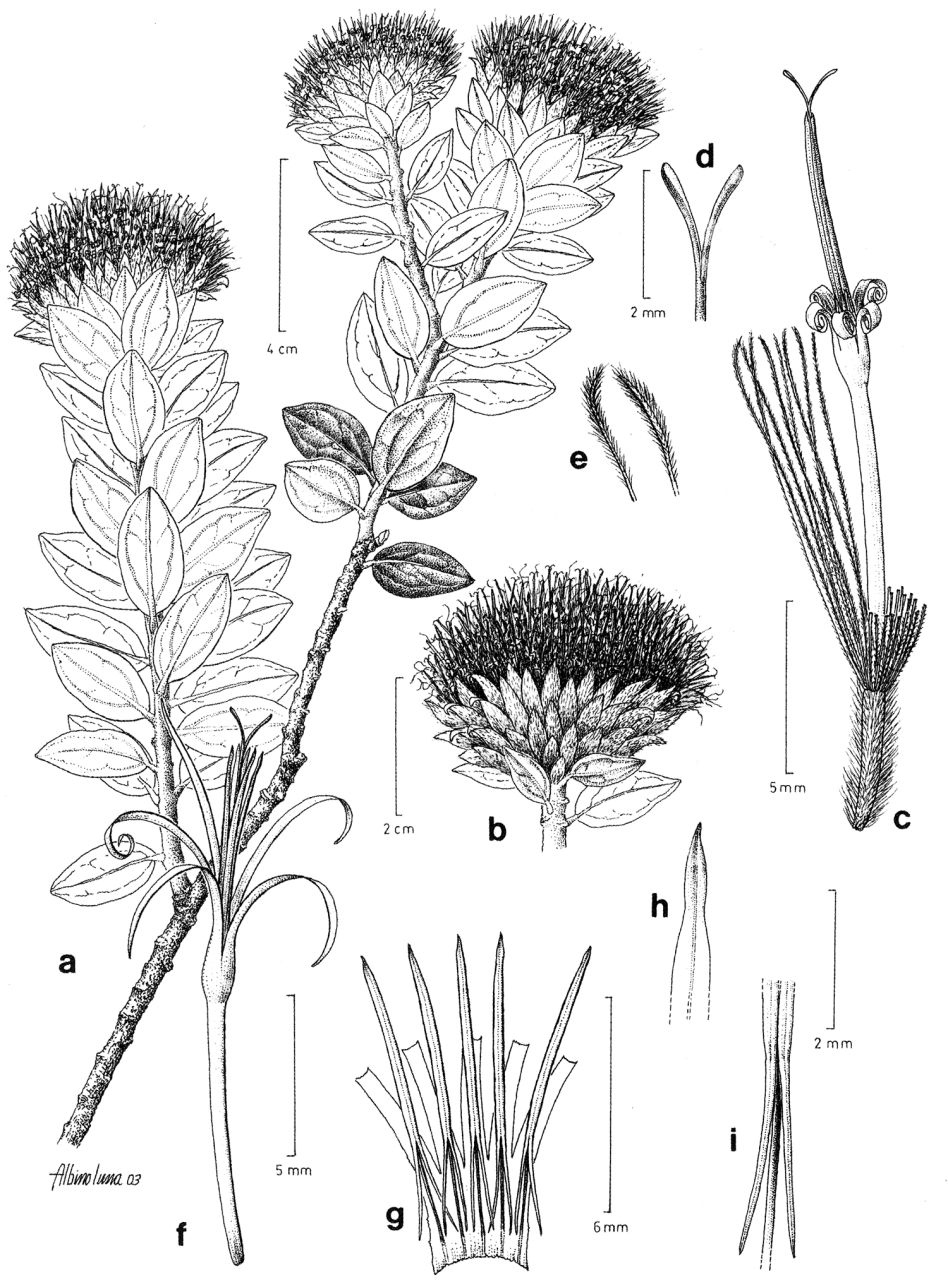
Drawing of *Nahuatlea hiriartiana*. **A** Habit **B** Head **C** Flower with achene **D** Style **E** Apex of pappus bristles **F** Flower **G** Anthers **H** Anther apex **I** Anther tails. [Modified from González-Medrano et al. 2004.]

#### Distribution.

Mexico: only known from two collections both in Hidalgo.

### 2. Nahuatlea magna

Taxon classificationPlantaeAsteralesCompositae

(M.C. Johnst. ex Cabrera) V.A.Funk comb. nov.

urn:lsid:ipni.org:names:60475587-2

Fig. 5

Gochnatia magna M.C. Johnson ex Cabrera, Revista Mus. La Plata, Secc. Bot., 12(66): 147–150. 1971.

#### Type.

Mexico. Tamaulipas: half a mile east of Nuevo Morelos, 25 Oct 1959, *J. Graham & M.C. Johnston 4485* (Holotype: MEXU 01220108; Isotype: TEX 00374383).

#### Description.

*Shrub or small tree* 2.5– 4.5 m tall; *leaves* chartaceous, blades 5–10 × 4.1–6.2 cm, widely elliptic, glabrescent adaxially, wooly abaxially; *heads* short pedunculate in clusters of 3–5 at the apex of branches, surrounded by leaves, few clusters per plant; *involucre* campanulate (especially at early flowering), 13–15 mm tall × 10–12 mm wide, bracts in 5–7 series, woolly adaxially; *flowers* ca. 50 per head; *corollas* yellow, 12 mm long; *anther* base caudate, tails ca. 1 mm long, entire; *pappus* ca. 9 mm long, bristles uniseriate, broadening, flattened, and somewhat darker at the apex.

**Figure 5.**
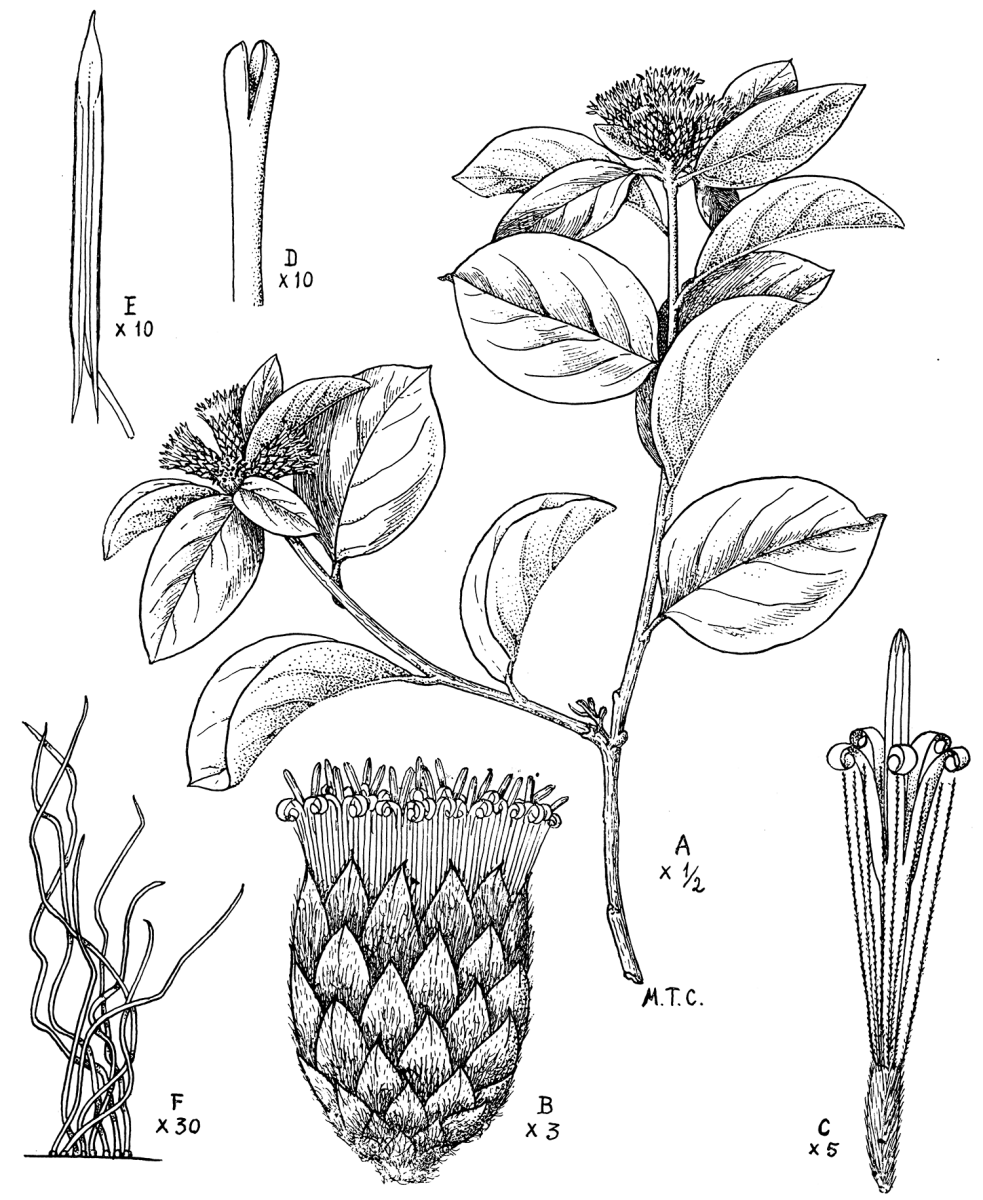
Cabrera’s drawing of *Nahuatlea magna*. **A** Habit **B** Head **C** Flower **D** Style **E** Anther **F** Pubescence. [Modified from Cabrera 1971.]

#### Distribution.

Mexico: Tamaulipas and San Luis Potosi.

### 3. Nahuatlea purpusii

Taxon classificationPlantaeAsteralesCompositae

(T.S. Brandegee) V.A.Funk comb. nov.

urn:lsid:ipni.org:names:77174064-1

Fig. 6

Gochnatia purpusii T.S. Brandegee, Zöe 5(11): 240. 1906.

#### Type.

Mexico. Puebla: Tehuacan, 1800 m, June 1905, *C.A. Purpus 1164* (Holotype: UC 91904; Isotypes: BM 000947904, F 0050268, GH 00008382, NY 00169558, RSA 0001214). [Specimens at P (P00703338 & P00703339) and E (E00413001) have the correct collecting number but incorrect dates and may or may not be type material; Cabrera (1971) cites isotypes at G, MO, and MEXU that are not found in JSTOR-GP. MO has confirmed that the specimen is not in their collection but there is no information on the others.

#### Description.

*Shrub* of unknown size (one isotype has what appears to be “5–6 m” written on the label); *leaves* coriaceous, petiole minute (1–2 mm), blade 1.8–3.0 × 0.5–1.4 cm, elliptical or slightly lanceolate or oblanceolate, glabrescent adaxially, white flocculent-tomentose abaxially; heads sessile, in few clusters of 8–15, clusters all at apex of branches; *involucre* campanulate (especially at early flowering), ca. 10 mm tall × 5–6 mm wide, bracts in 5–6 series, densely wooly abaxially; *flowers* 12–18 per head; corollas yellowish, ca. 8 mm long; *anther* base caudate, tails ca. 1 mm long, fimbrillate; *pappus* ca. 7.5 mm long, bi-seriate with slender bristles.

**Figure 6.**
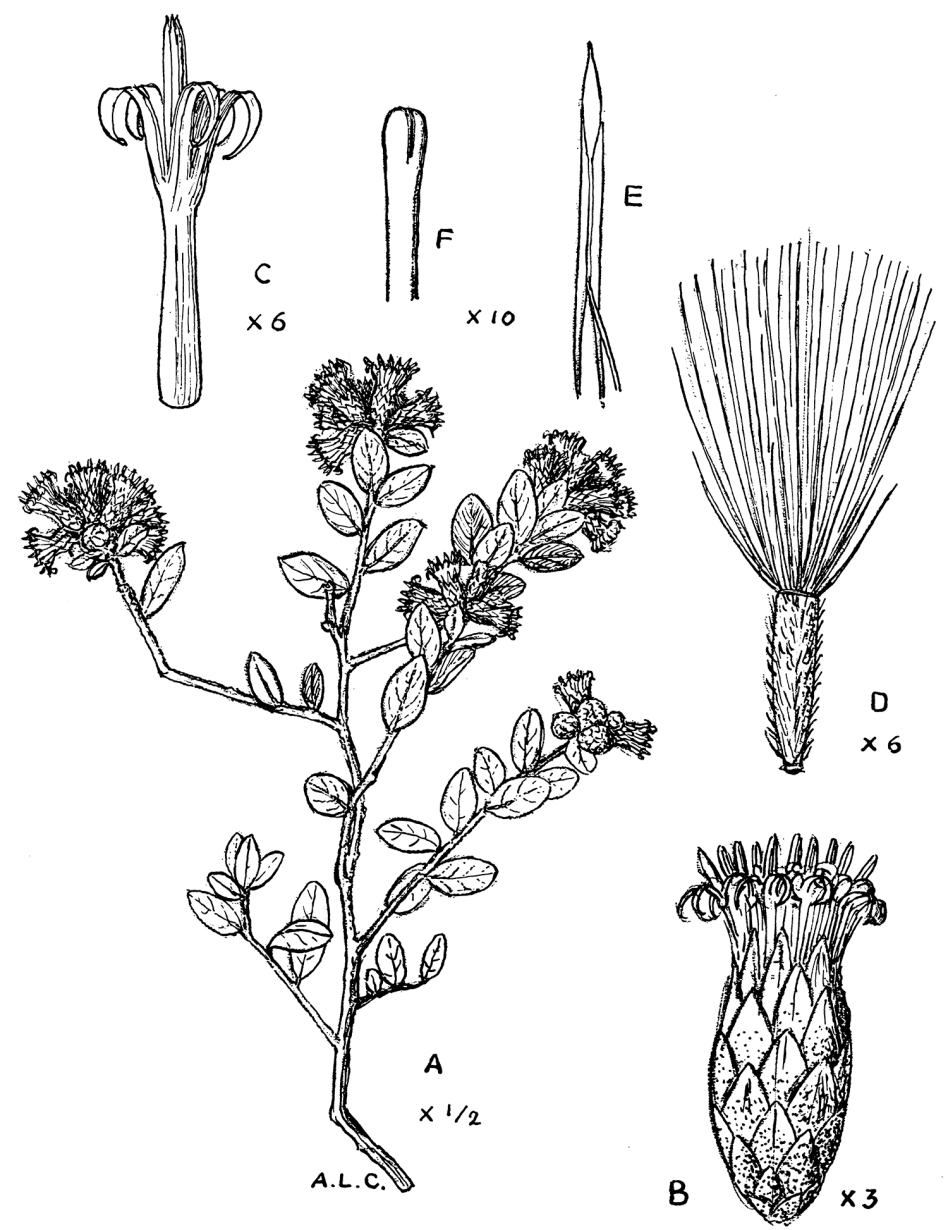
Cabrera’s drawing of *Nahuatlea purpusii*. **A** Habit **B** Head **C** Style **D** Achene with pappus **E** Anther **F** Style. [Modified from Cabrera 1971.]

#### Remarks.

Cabrera (1971) and Jervis (1954) disagree somewhat on the size of the leaf blade: Cabrera lists it as 18–30 mm × 5–14 mm and Jervis has 2.0–2.5 cm × 0.5–1.0 cm. Our measurements, taken from the Holotype, fall within those given by Jervis, however, the isotypes may show the greater range given by Cabrera.

#### Distribution.

Mexico: known only from four collections all from Puebla.

### 4. Nahuatlea arborescens

Taxon classificationPlantaeAsteralesCompositae

(Brandegee) V.A.Funk comb. nov.

urn:lsid:ipni.org:names:77174065-1

Fig. 3B

Gochnatia arborescens Brandegee, Zoë 5(9): 163. 1903.

#### Type.

Mexico. Baja California: Cañón de Santa María, El Juste, 1 Mar 1898, *C.A. Purpus s.n.* (Holotype: UC 91900; possible Isotypes: K 000502538; GH 00008379; US 00119526).

#### Note.

Determining what may or may not be type material is difficult; all proposed types are *Purpus s.n.* collections but there are different collection dates and locality information. The Holotype is one of two different collections mounted on the same sheet one of which is annotated as “n. sp.” and “type”. That specimen also has a hand-written note that says “The year doubtless 1901 RM” indicating that the date on the sheet January-March 1898 is not correct. The information found on the three possible isotypes is nearly identical (Lower California, San Felipe, Cape Region Lower California, Jan-Mar 1901) but the information is different from the locality information on the Holotype. All of the “type material” specimens are the same species so there is little doubt that this is the correct name for this entity but someone more familiar with the work of Purpus (who seems to have confusing dates and numbers) and Brandegee will need to investigate this further to determine if the three listed specimens are actually isotypes.

#### Description.

*Tree* 3–8 m tall, trunk 20–30 cm in diameter; *leaves* chartaceous, blades 3.5–6.5 cm × 2.5–4.5 cm, ovate to elliptic, cuneate or rounded at base, glabrescent on both faces; heads short-pedunculate, solitary or more usually in loose clusters of 2–20 at apex of branches, few clusters per plant; *involucre* cylindrical to narrowly obconic depending on age, 10–15(20) mm tall × 6–8 mm wide at anthesis, bracts in 8–10 series, glabrous; *flowers* 13–20 per head; corollas yellowish, 12 mm long; *anther* base caudate, tails ca. 3 mm long, entire; *pappus* ca. 11 mm long, bristles slender, biseriate.

#### Remarks.

Cabrera (1971) and Jervis (1954) disagree somewhat on the size of the leaf blade: Cabrera lists it as 35–65 mm × 25–45 mm and Jervis has 5 cm × 3–4 cm. Specimens available to us agree with the range given by Cabrera.

#### Distribution.

Mexico: known only from Baja California Sur.

### 5. Nahuatlea obtusata

Taxon classificationPlantaeAsteralesCompositae

(S.F. Blake) V.A.Funk comb. nov.

urn:lsid:ipni.org:names:77174066-1

Figs 3D , 7

Gochnatia obtusata S.F. Blake, Contributions from the United States National Herbarium 22: 652. 1924.

#### Type.

Mexico. Puebla: dry limestone hills at Tehuacan, 20 Dec 1895, *C.G. Pringle 6253* (Holotype: US 00119530 [254705]; Isotypes: A 00008380, BM 000947903, BR 0000005318124, CM 2403, E 00413002, JE 00000693, K 000502539, GH 00008381, MEXU 01220107, MO 1544305, NDG 63791, NY 00169557, P 00703351, P 00703352, PH 00025961, S 10-11705, TEX 00000454, US 01100608). [Cabrera (1971) mentions additional specimens at F, G, M, UC, and W but these were not found in JSTOR-GP.]

#### Description.

*Shrub* 1.0–1.5(–2.6) m tall; *leaves* coriaceous, (4.5)5–8(20) × 3–5 mm, broadly elliptic, apex rounded to obtuse, rounded at base, glabrous adaxially, cinereous-tomentose abaxially; heads short-pedunculate (ca. 2 mm long), in clusters of 10–20 heads, at apices of branches; *involucre* narrowly obconic, 6–8 mm tall × 2.8–3.2 mm wide, bracts in ca. 5 series, glabrous; *flowers* 5–6 per head; corollas white, 6.5–7.5 mm long; *anther* base caudate, tails ca. 2 mm long, fimbrillate; *pappus* 6.0–7.5 mm long, bristles biseriate, broadening slightly at the tips.

Jervis (1954) said that the leaf length is “rarely up to 2 cm” and the width is “less than 1 cm” and Cabrera (1971) lists the length as 4–20 × 3–13 mm. Both seem to be correct; we found only one specimen that had leaves 20 mm long and all but two were less than 1 cm.

**Figure 7.**
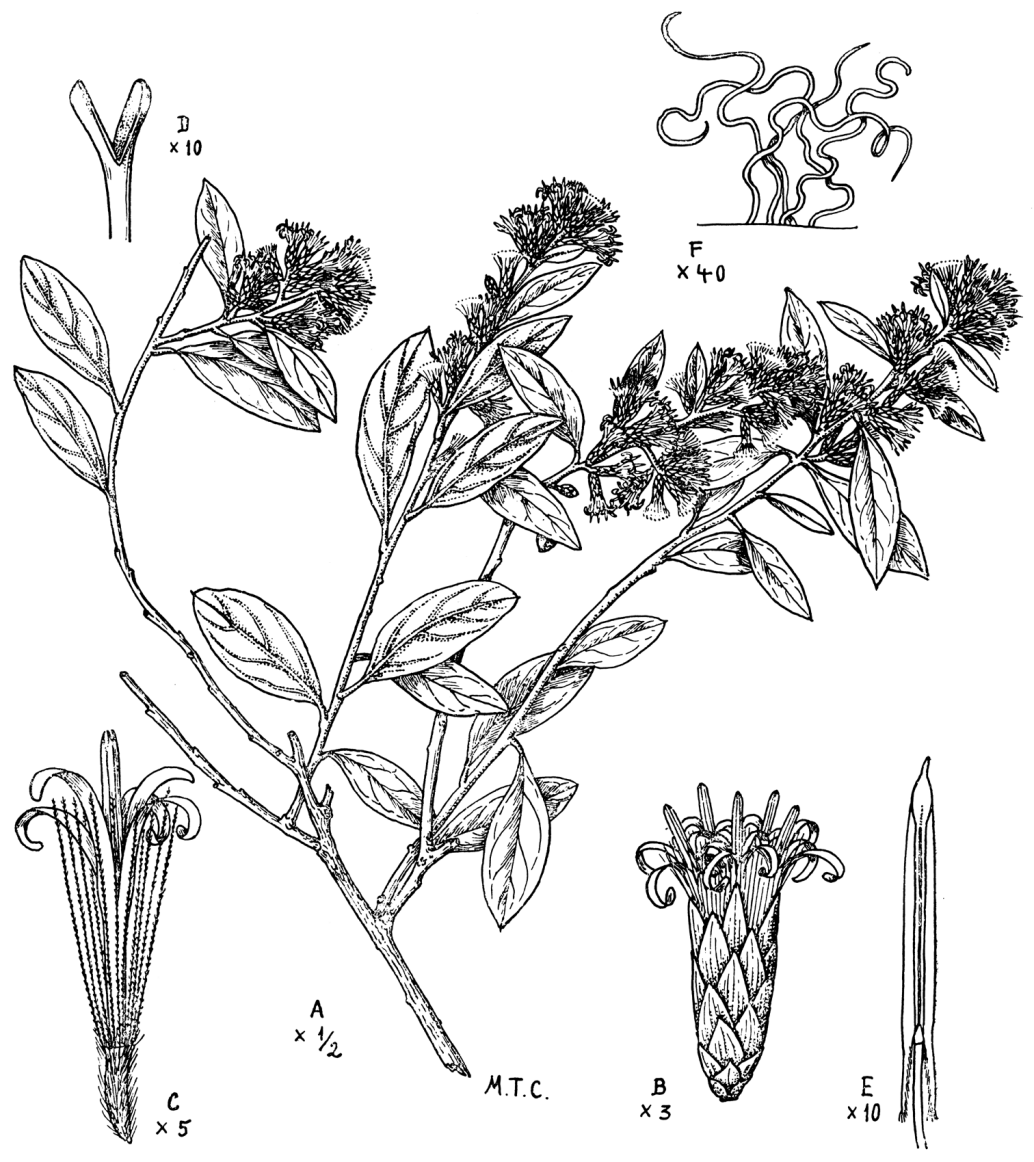
Cabrera’s drawing of *Nahuatlea hypoleuca*. **A** Habit **B** Head **C** Floret **D** Style **E** Anther **F** Pubescence. [Modified from Cabrera 1971.]

#### Distribution.

Mexico: Puebla and Oaxaca.

#### Remarks.

*Gochnatia obtusata* was described by S. F. Blake (1924) and accepted as a good species by Jervis (1954). Cabrera placed it as a subspecies of *G. hypoleuca* as did Freire et al. (2002). The difference of opinion is based on the importance one gives to the characters that separate the two entities. Blake stated that *G. hypoleuca* could be separated from *G. obtusata* by the following characters: leaves chiefly elliptic, apex acute or acutish and mucronate, 20–50 mm × 8–15 mm; involucre 5–6 mm tall, phyllaries obtuse to acute or obtusely acuminate. We have added some additional characters: shrub or small tree 3–7 m; heads arranged in short clusters or panicles; involucre with bracts in 3–5 series; corolla 10–12 mm long; style branches 0.7–1 mm long; pappus up to 6.5 mm. In contrast, *G. obtusata* has the following: leaves broadly elliptic, apex broadly rounded to obtuse, not mucronate, 4.5–20 mm × 3–13 mm; involucre 6.5–7.5 mm tall, involucral bracts acute to sharply acuminate; style branches 0.5–0.7 mm long. Additional characters for *G. obtusata* include: shrub of 1.0–2.6 m; heads glomerate at tips of branches and in clusters of 1–several in the subterminal axils; involucre with bracts in 5-6 series; corollas 6.5–7.5 mm long; pappus up to 7.5 mm long. In the field the difference is striking with the larger more robust *G. hypoleuca* contrasting with the smaller, more compact *G. obtusata*. Therefore we have chosen to recognize *G. obtusata* as a separate species.

### 6. Nahuatlea hypoleuca

Taxon classificationPlantaeAsteralesCompositae

(DC.) V.A.Funk comb. nov.

urn:lsid:ipni.org:names:60475588-2

Figs 3A , 8

Moquinia hypoleuca DC., Prodromus 7(1): 23. 1838.
Gochnatia hypoleuca (DC.) A. Gray, Proceedings of the American Academy of Arts and Sciences 19: 57. 1883.

#### Type.

Mexico. Neuvo León: Monterrey, January 1828, *Berlandier 1391* (Holotype: G 00223915; Isotypes: BM 000947902, GH 00010616, K 000502540, MO 100221306, NY 00230667, P 00703318, P 00703319, P 00703320, US 00119521). [There is a second specimen at NY that is a possible isotype NY 00230666; the specimen from HAL 0112991 may be an isotype but the dates don’t match and the number is listed at “1391 s.n.”]

#### Description.

*Shrub* or small tree, 2–5(–7) m tall; *leaves* coriaceous, 2–5 × 0.9–1.5 cm, narrowly elliptic, apex obtuse, mucronate, attenuate at base, glabrous adaxially, cinereous-tomentose abaxially; heads sessile or very short pedunculate, in clusters of 5–15 heads at apices and axils of branches, many clusters per plant; *involucre* narrowly obconic, 4–7 mm tall × ca. 3 mm wide, bracts in ca. 3–5 series, ciliolate-tomentose on the margins but otherwise glabrous; *flowers* 5–7 per head; *corollas* white, 10–12 mm long; *anther* base caudate, tails ca. 1 mm long, fimbrillate; *pappus* ca. 6.5–7.5 mm long, bristles biseriate, of various lengths and broadening slightly at the tips.

**Figure 8.**
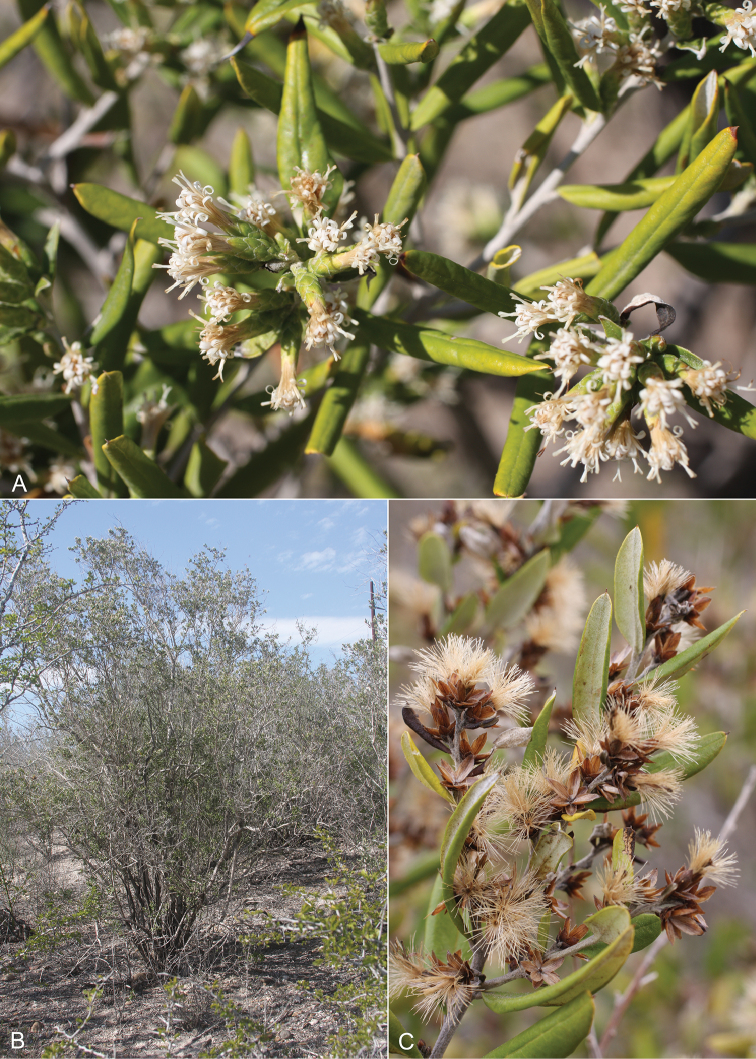
Photos of *Nahuatlea hypoleuca* from southern Texas. Habit photo from La Puerta, Texas; Fruiting photo from Vaquillas Road, Texas; Flowers from Yturria Brush, Texas. [photos by T. F. Patterson.]

**Figure 9.**
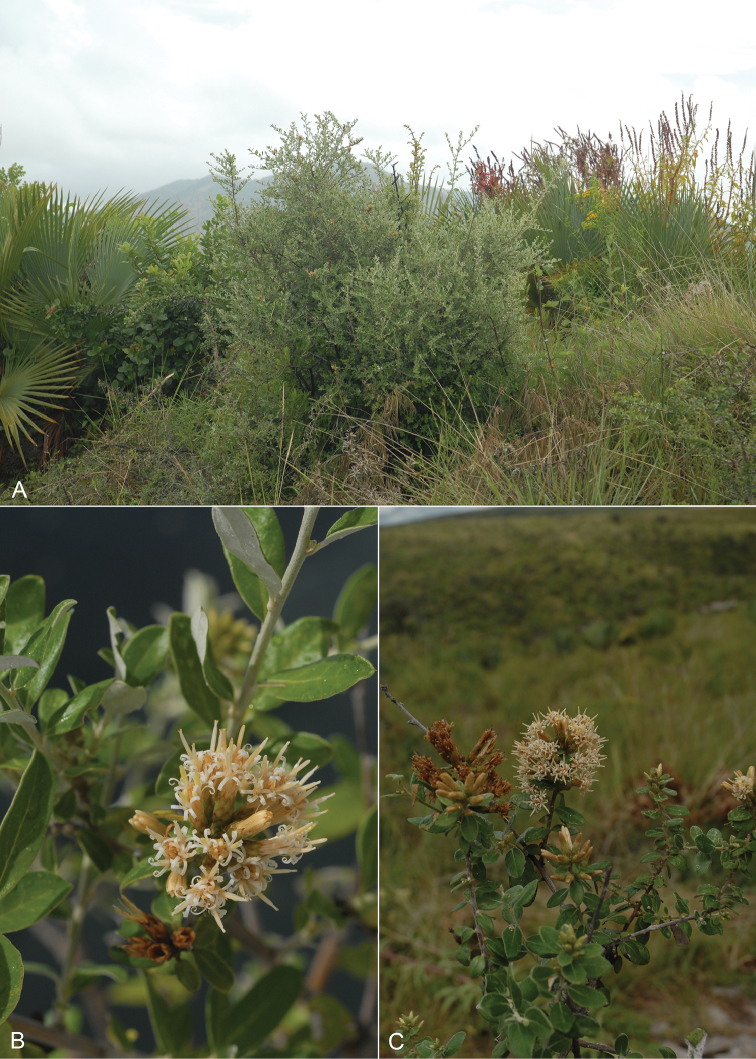
Photos of *Nahuatlea obtusata*, all from Oaxaca, Mexico, showing habit and flowering heads of different ages. [photos by V. A. Funk.]

#### Distribution.

Mexico: Coahuila, Nuevo León, Tamaulipas, Durango, Zacatecas, San Luis Potosi, Queretaro, Hidalgo, and Michoacán. United States: Arizona, Texas.

#### Remarks.

According to the Texas A&M University website (http://aggie-horticulture.tamu.edu/ornamentals/nativeshrubs/gochnatiahypole.htm) the common names for this shrub in Texas are Chomonque and Ocote. The website goes on to say that it is an “attractive, little-known shrub native to extreme South Texas, Chomonque flowers in the winter and has striking bi-colored leaves, very dark green on top and white and feltish underneath. The white flowers that appear from November to February are weakly fragrant and attract bees and many species of butterflies. It grows on gravel and caliche in South Texas shrub lands, and is extremely drought and heat tolerant. A specimen at the San Antonio Botanical Gardens has proved cold hardy and evergreen, but its cold-hardiness farther north is untested.” The website lists the USDA hardiness zone 9.

### 7. Nahuatlea smithii

Taxon classificationPlantaeAsteralesCompositae

(B.L. Robinson & Greenm.) V.A.Funk comb. nov.

urn:lsid:ipni.org:names:77174067-1

Fig. 3C

Gochnatia smithii B.L. Robinson & Greenm., Proc. Amer. Acad. Arts 32: 50. 1896.

#### Type.

Mexico. Oaxaca: On the hills of Cuicatlan, April 1895, *L.C. Smith 363* (Lectotype: K designated by Cabrera, 1971: 140; Isolectotypes: GH 00008383, MEXU 00525748).

#### Description.

*Shrub* of undetermined size; *leaves* clustered near the ends of the branches, sub-coriaceous, 3–5 × 0.8–1.8 cm, ovate, obtuse, cuneate at base, glabrescent adaxially, cinereous-tomentose abaxially; heads slender, short-pedunculate, in dense clusters at the ends of branches, sometimes several clusters in a dense panicle, many clusters per plant; *involucre* narrowly obconic, 6–8 mm tall × ca. 3 mm wide, bracts in 5–7 series, ciliolate on the margins otherwise glabrous; *flowers* (3–)5–7 per head; *corollas* white, 8.5–9.0 mm long; *anther* base caudate, tails ca. 1.5 mm long, entire; *pappus* ca. 6.5–7.5 mm long, bristles biseriate, not obviously broadened at tips.

#### Remarks.

According to Jervis (1954) the “…small branches are less wooly than most other species.”

#### Distribution.

Mexico: known only from Oaxaca.

## Supplementary Material

XML Treatment for Nahuatlea

XML Treatment for Nahuatlea hiriartiana

XML Treatment for Nahuatlea magna

XML Treatment for Nahuatlea purpusii

XML Treatment for Nahuatlea arborescens

XML Treatment for Nahuatlea obtusata

XML Treatment for Nahuatlea hypoleuca

XML Treatment for Nahuatlea smithii
